# Aqueous Synthesis of 1-*H*-2-Substituted Benzimidazoles via Transition-Metal-Free Intramolecular Amination of Aryl Iodides

**DOI:** 10.3390/molecules171112506

**Published:** 2012-10-24

**Authors:** Chunxia Chen, Chen Chen, Bin Li, Jingwei Tao, Jinsong Peng

**Affiliations:** 1 Department of Chemistry and Chemical Engineering, College of Science, Northeast Forestry University, Harbin 150040, China; 2 Post-Doctoral Mobile Research Station of Forestry Engineering, Northeast Forestry University, Harbin 150040, China

**Keywords:** benzimidazoles, aqueous synthesis, *N*-arylation, transition-metal-free conditions, aryl iodides

## Abstract

A straightforward method has been developed for the synthesis of the benzimidazole ring system through a carbon-nitrogen cross-coupling reaction. In the presence of 2.0 equiv. of K_2_CO_3_ in water at 100 °C for 30 h, the intramolecular cyclization of *N*-(2-iodoaryl)benzamidine provides benzimidazole derivatives in moderate to high yields. Remarkably, the procedure occurs exclusively in water and doesn’t require the use of any additional reagent/catalyst, rendering the methodology highly valuable from both environmental and economical points of view.

## 1. Introduction

**Figure 1:**
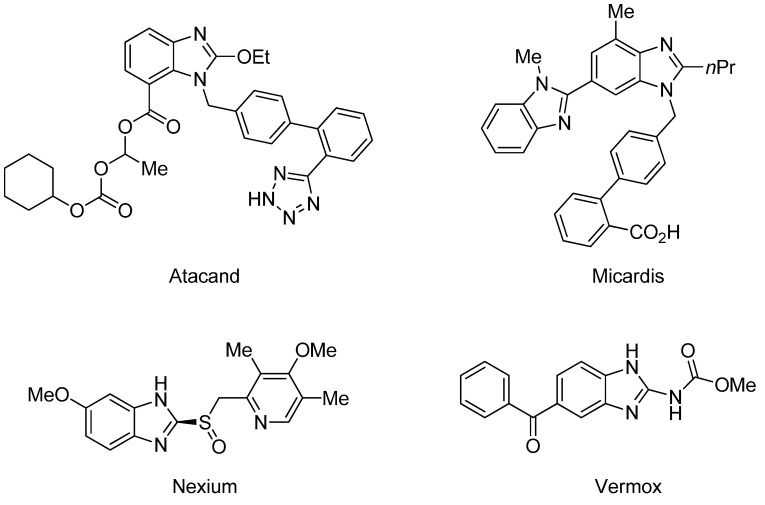
Structures of some pharmacologically important benzimidazoles.

Benzimidazoles are an important class of heterocycles that are frequently used in drug and agrochemical discovery programs. For examples, the benzimidazole core structure is found in a variety of commercial drugs such as Atacand, Nexium, Micardis, Protonix, and Vermox (Figure 1). Recent medicinal chemistry applications of benzimidazole analogs include antibacterial and antifungal agents [1,2,3], anthelmintic agents [4], HIV-1-induced cytopathic inhibitor [5], anti-inflammatory and antiulcer agents [6], cytotoxic and antitumor agents [7,8], DNA binding agents [9], enzyme and receptor agonists or antagonists [10]. Other applications of benzimidazoles include their use as organic ligands [11,12], fluorescent whitening agent dyes [13] and functional materials [14,15]. Therefore, the construction of these heterocycles has always been of great interest to organic and medicinal chemists and has consequently received much attention [16].

**Scheme 1:**
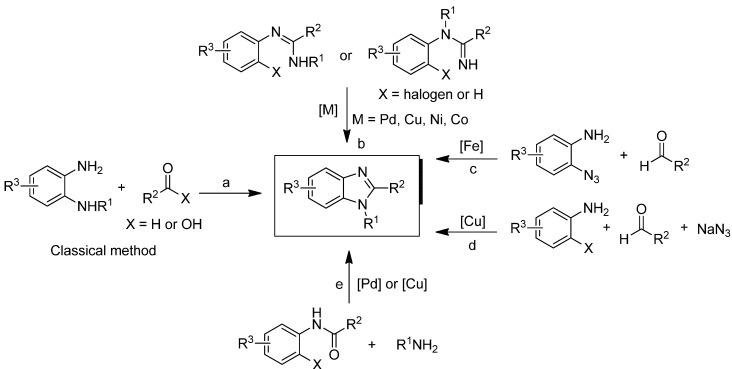
Available methods to assemble benzimidazole derivatives.

The classical and most common methods to assemble benzimidazoles involve the condensation of benzene-1,2-diamines with aldehydes, carboxylic acids, or their derivatives (Scheme 1, route a) under strong acid/high temperature conditions or using a stoichiometric oxidant [17,18,19,20]. Although these transformations are widely used owing to their inherent simplicity, this method is restricted to the available starting materials and involves harsh reaction conditions [17,18,19,20]. Furthermore, this methodology is not suitable for the regioselective synthesis of *N*-substituted benzimidazoles, as both syntheses result in regioisomers and disubstituted products from the 1,2-diaminoarene. To circumvent these restrictions, the transition-metal-catalyzed amination approach is a viable strategy to construct the benzimidazole ring regiospecifically. Among the different catalysts, palladium- [21,22,23,24,25], copper- [26,27,28,29,30,31,32,33], nickel- [34], iron- [35], and cobalt-based [36] complexes are generally employed for this coupling reaction (Scheme 1, routes b–e). Despite these recent advances, transition-metal-catalyzed methods are often expensive and require especially designed ligands. Another disadvantage is the need to find ways to remove metal-related impurities from products, an important issue in the synthesis of pharmaceutical compounds.

Transition-metal-free *N*-arylation reactions [37,38,39,40,41,42,43,44] are also known to occur either by nucleophilic aromatic substitutions [45] or aryne-type intermediates [46,47,48,49,50] in the presence of a base. The former usually requires dipolar aprotic solvents (such as DMF, NMP and DMSO) and sometimes high reaction temperatures; the latter method requires strongly basic reaction conditions (generally potassium amide in liquid ammonia or *n*-BuLi in hexane). Both synthetic procedures have some drawbacks: harsh reaction conditions, inconvenient handling and workup, or a relatively narrow scope of substrates. Green reaction conditions in synthetic processes have been advocated, and extensive efforts have been devoted to finding sustainable reaction media. Notably the use of water as solvent has attracted much attention in recent years [51,52,53,54]. In parallel with our efforts to develop metal-free synthetic protocols for the production of pharmaceutical and agrochemical heterocyclic compounds [55,56], we envisaged the application of more sustainable protocol to the aqueous synthesis of the benzimidazole framework under transition metal-free conditions.

**Scheme 2:**
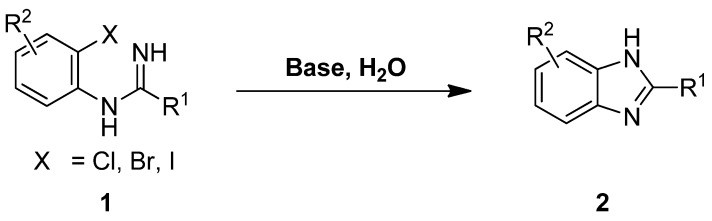
Proposed approach to the synthesis of benzimidazoles **2**.

As shown in Scheme 2, we propose the synthesis of benzimidazole derivatives **2** through a direct base-mediated intramolecular *N-*arylation reaction in water, starting from the corresponding *N*-(2-haloaryl) amidine **1**.

*N*-(2-Halophenyl) benzamidines **1a**–**a"** were selected as model substrates for this *N*-arylation reaction. In fact, our recently reported copper-catalyzed amination [28] showed that 2-iodoarylbenzamidine **1a** (with a concentration of 0.67 mol/L on a 1 mmol scale) can be transformed into the corresponding product in 19% yield with K_2_CO_3_ in water at 100 °C for 30 h. The use of Cu_2_O/DMEDA as the catalyst could efficiently promote this transformation giving 98% yield. Based on the above observations, we wondered whether this copper-free chemical reaction can be improved by changing heterogeneity, oil-water interface, and modes of aggregation “on” the surface of water or in water [57,58]. Further investigations showed that using a relatively low concentration (about 0.1 mol/L on a 0.25 mmol scale), benzimidazole can be obtained in moderate to high yields with vigorous stirring in water.

## 2. Results and Discussion

Optimization of other reaction conditions such as base, temperature and time is shown in Table 1. At ﬁrst, the control experiment of **1a** was examined in the absence of a base (entry 1, Table 1), and the desired product was not observed. The intramolecular carbon-nitrogen cross-coupling reaction of *N*-(2-iodophenyl)benzamidine (**1a**) using potassium carbonate (K_2_CO_3_, 2.0 equiv.) as the base in water at 100 °C for 30 h was then examined. To our delight, benzimidazole **2a** was smoothly obtained in 80% yield (entry 2, Table 1).

**Table 1:** Optimization of base-mediated intramolecular C–N cross-coupling of benzamidine **1a**–**c** in water ^[^^a^^]^. 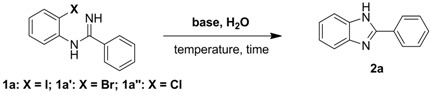

Entry	Substrate	Base	Temperature (°C)	Time (h)	Yield (%) ^[^^b^^]^
1	**1a**	―	100	30	0
2	**1a**	K_2_CO_3_	100	30	80
3	**1a**	KOH	100	30	63
4	**1a**	K_3_PO_4_	100	30	trace
5	**1a**	NaOH	100	30	0
6	**1a**	NaHCO_3_	100	30	0
7	**1a**	Na_2_CO_3_	100	30	0
8	**1a**	Cs_2_CO_3_	100	30	84
9	**1a**	Et_3_N	100	30	0
10	**1a**	Pyridine	100	30	0
11 ^[^^c^^]^	**1a**	K_2_CO_3_	80	30	trace
12	**1a**	K_2_CO_3_	90	30	60
13	**1a**	K_2_CO_3_	100	20	50
14	**1a**	K_2_CO_3_	100	48	74
15 ^[^^d^^]^	**1a**	K_2_CO_3_	120	30	78
16 ^[^^d^^]^	**1a**	K_2_CO_3_	150	30	66
17	**1a'**	K_2_CO_3_	100	30	0
18	**1a''**	K_2_CO_3_	100	30	0

^[a^^]^ The reaction was carried out with *N*-(2-halophenyl)benzamidine (0.25 mmol) and base (0.5 mmol) in water (2.0 mL) with vigorous stirring at 80–150 °C for 20–48 h; ^[b^^]^ Isolated yield after column chromatography; ^[c^^]^ Complete recovery of starting material; ^[d^^]^ Decomposition product *o*-bromoaniline was also obtained under the given reaction conditions.

Recent research has revealed that metal impurities in commercially available reagents might potentially affect their reactions [59,60,61,62]. To eliminate this possibility, different sources of K_2_CO_3_ and puriﬁed K_2_CO_3_ with high purities (99.9%) were used with new glassware, and metal reagents were avoided in synthetic steps wherever possible, and almost the same yields were obtained. Furthermore, based on the data from entries 2 to 10 in Table 1, we concluded that the presence of trace metal impurities weren’t involved in this carbon-nitrogen bond formation reaction [63]. The nature of base was very important to the reaction outcome. KOH and Cs_2_CO_3_ were also effective in promoting this C–N bond formation in water, and the following yields were obtained: 63% (KOH) and 84% (Cs_2_CO_3_). Surprisingly, other bases such as NaOH, NaHCO_3_, K_3_PO_4_, Na_2_CO_3_, Et_3_N and pyridine gave no product. The reactions performed at 100 °C gave the best result, because at lower temperature the conversions remained incomplete (entries 11 and 12, Table 1), at higher temperature the undesired decomposition of substrate to *o*-iodoaniline happened (entries 15 and 16, Table 1). The *ortho-*substituted halogen on the aniline moiety was very important to this intramolecular carbon-nitrogen cross-coupling reaction. Aryl chloride and aryl bromide, which were expected to be more reactive than their iodo analogues in a substitution reaction proceeding by the S_N_Ar mechanism [64,65], gave no product. Obviously an aromatic nucleophilic substitution process is inconsistent with our experimental results (entries 17 and 18, Table 1), so this reaction presumably occurred by an aryne-type intermediate in the presence of a base.

With the optimized reaction conditions in hand, the generality of the aniline moiety in the amination process was explored first. As shown in Table 2, (*o*-iodoaryl)benzamidines can smoothly be converted to the desired products in moderate to high yields, however, the use of aryl bromides to effect such transformations afforded none of the desired products (entries 3 and 9, Table 2). For aryl iodides, a variety of substituents such as F, Cl, Br, Me and MeO can be used. It is worth noting that reaction conditions compatible with C–Br or C–Cl combinations are particularly appealing, since these substituents offer great opportunity for further synthetic manipulations (entries 4 and 21, Table 2). 3-Iodo-2-aminopyridine substrate **1g** can be transformed into the corresponding benzimidazole in 44% yield (entry 8, Table 2), however, 2-iodo-3-aminopyridine substrate **1h** gave no product (entry 10, Table 2) that probably attributed to failure to generate an aryne intermediate by ortho-deprotonations followed by iodide elimination. These results as well as the order of reactivity of aryl halides (entries 2, 17 and 18. Table 1) further pointed to the involvement of aryne-type intermediates.

The scope and limitation of the nitrile moiety were next studied (Table 3). Obviously, the electronic nature of the benzonitrile motifs had a great effect on the yields. Substrates bearing various electron-donating substituents such as Me–, MeO– and Me_2_N– can be converted smoothly into the desired products in moderate to high yields (entries 1–6, Table 3). Furthermore, the steric hindrance of *ortho* substituents on the benzonitrile moiety seemed not to hamper *N*-arylation reaction, the benzimidazoles could be obtained in similar yields (entries 1–4, Table 2). However, the presence of relatively electron-withdrawing or stronger electron-withdrawing functional groups completely held back intramolecular amination process. Other electron-rich aromatic and heteroaromatic substrates such as **1q**, **1r** and **1s** could be efﬁciently transformed into the corresponding benzimidazoles in satisfactory yields (entries 9–11, Table 3). In addition, *N′*-phenylated alkylamidine substrate **1u** could also be converted to the desired product **2u** under these conditions (entry 13, Table 3). In contrast to electron-rich aromatic substituents, *N*-(2-iodophenyl)amidine with an aliphatic functional group (Me–) provided a trace amount of the product (entry 12, Table 3), the most of the starting materials were unchanged and recovered from the reaction mixture.

**Table 2:** Direct weak base-mediated synthesis of 2-phenylbenzimidazole derivatives in water ^[^^a^^]^. 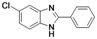

Entry	Substrate	Product	Yield (%) ^[^^b^^]^
1	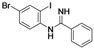 **1a**	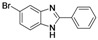 **2a**	80
2	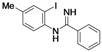 **1b**	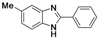 **2b**	77
3^c^	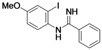 **1b**'	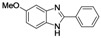 **2b**	0
4	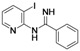 **1c**	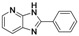 **2c**	66
5	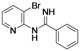 **1d**	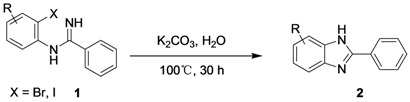 **2d**	54
6	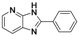 **1e**	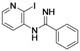 **2e**	67
7	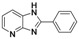 **1f**	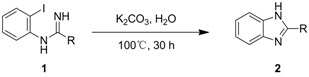 **2f**	67
8	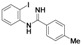 **1g**	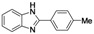 **2g**	44
9 ^[c]^	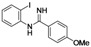 **1g'**	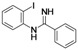 **2g**	0
10 ^[c]^	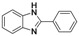 **1h**	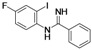 **2h**	0

^[a^^]^ Reaction conditions: 1.0 equiv. of *N*-(2-haloaryl)benzamidine (0.25 mmol) and 2.0 equiv. of K_2_CO_3_ in water (2.0 mL) at 100 °C with vigorous stirring for 30 h; ^[b^^]^ Isolated yield after column chromatography; ^[c^^]^ Complete recovery of starting material.

**Table 3:** Synthesis of 2-arylbenzimidazole derivatives in water ^[^^a^^]^. 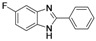

Entry	Substrate	Product	Yield (%) ^[^^b^^]^
1	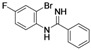 **1i**	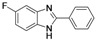 **2i**	60
2	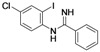 **1j**	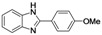 **2j**	63
3	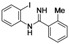 **1k**	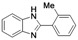 **2k**	58
4	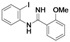 **1l**	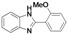 **2l**	64
5	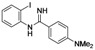 **1m**	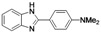 **2m**	70
6	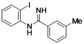 **1n**	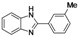 **2n**	50
7 ^[c]^	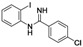 **1o**	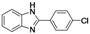 **2o**	0
8 ^[c]^	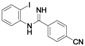 **1p**	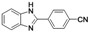 **2p**	0
9	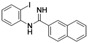 **1q**	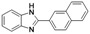 **2q**	48
10	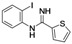 **1r**	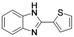 **2r**	60
11	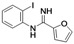 **1s**	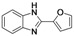 **2s**	48
12 ^[c]^	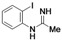 **1t**	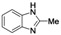 **2t**	0
13	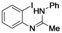 **1u**	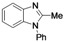 **2u**	33

^[a^^]^ Reaction conditions: 1.0 equiv. of *N*-(2-iodophenyl)amidine (0.25 mmol) and 2.0 equiv. of K_2_CO_3_ in water (2.0 mL) at 100 °C with vigorous stirring for 30 h; ^[b^^]^ Isolated yield after column chromatography; ^[c^^]^ Complete recovery of starting material.

## 3. Experimental

### 3.1. General

Chemicals and solvents were all purchased from commercial supplies and used without further purification. Amidines were prepared through the addition of an aniline to a nitrile according to known procedures [20,21,22,23,24]. Silica gel (100 mesh) was used for chromatographic separation. Silica gel G was used for TLC. Petroleum ether refers to the fraction boiling between 60 °C and 80 °C. All reactions were carried out in dried glassware. ^1^H-NMR spectra were recorded on a Bruker-400 MHz spectrometer and ^13^C-NMR spectra were recorded at 100 MHz using tetramethylsilane (TMS) as the internal standard in DMSO-*d*_6_. Chemical shifts (δ) are given in parts per million (ppm) downfield relative to TMS (^1^H-NMR: TMS at 0.00 ppm, DMSO at 2.50 ppm; ^13^C-NMR: DMSO at 40.0 ppm). Yields refer to isolated yields of compounds estimated to be >95% pure as determined by ^1^H-NMR. Melting points were determined by use of a Buchi melting point apparatus and not corrected. High-resolution mass spectra were recorded on a Bruker BIO TOF Q mass spectrometer.

### 3.2. Chemistry

#### 3.2.1. General Procedure for the Preparation of Benzimidazoles **2a–u**

A 10 mL Schlenk tube equipped with a magnetic stirring bar was charged with the (*o-*iodoaryl)-benzamidine substrate (0.25 mmol, 1.0 equiv.) and K_2_CO_3_ (69 mg, 0.5 mmol, 2.0 equiv.), then H_2_O (2.0 mL) was added via syringe at room temperature. The tube was sealed and put into a pre-heated oil bath at 100 °C for 30 h. The reaction mixture was cooled to room temperature, quenched with water (3 mL), and diluted with ethyl acetate (5 mL). The layers were separated and the aqueous layer was extracted with (2 × 5 mL) ethyl acetate. The combined organic extracts were dried over anhydrous sodium sulfate, filtered, and concentrated *in vacuo*. The crude product was then purified by flash chromatography on silica gel (H), eluting with 5–10% ethyl acetate/petroleum ether.

*2-Phenyl-1H-benzo[d]imidazole* (**2a**). White solid; m.p. 293–295 °C; yield: 80%. ^1^H-NMR: δ 12.92 (br s, 0.19H), 8.21–8.19 (d, 2H, *J* = 7.6 Hz), 7.69–7.67 (d, 1H, *J* = 6.8 Hz), 7.58–7.48 (m, 4H), 7.24–7.22 (d, 2H, *J* = 6.8 Hz). ^13^C-NMR: δ 151.55, 144.23, 135.30, 130.57, 130.32, 129.42, 126.89, 123.02, 122.15, 119.34, 111.75. HRMS-ESI (*m/z*): [M+Na]^+^ calcd. for C_13_H_10_N_2_Na 217.0742; found 217.0745.

*5-Fluoro-2-phenyl-1H-benzo[d]imidazole* (**2b**). White solid; m.p. 243–244 °C; yield: 77%. ^1^H-NMR: δ 13.06 (br s, 0.14H), 8.18–8.16 (d, 2H, *J* = 8.0 Hz), 7.69–7.31 (m, 5H), 7.12–7.04 (m, 1H). ^13^C-NMR: δ 160.15, 153.33, 140.91, 130.60, 130.28, 126.95, 120.19, 111.19, 110.44, 104.90, 98.28. HRMS-ESI (*m/z*): [M+Na]^+^ calcd. for C_13_H_9_FN_2_Na 235.0647; found 235.0649. 

*5-Chloro-2-phenyl-1H-benzo[d]imidazole* (**2c**). White solid; m.p. 209–211 °C; yield: 66%. ^1^H-NMR: δ 13.13 (br s, 0.18H), 8.20–8.18 (d, 2H, *J* = 7.2 Hz), 7.74–7.51 (m, 5H), 7.26–7.24 (d, 1H, *J* = 8.0 Hz). ^13^C-NMR: δ 152.55, 144.63 (142.48), 135.52 (133.60), 130.15, 129.56, 128.94, 126.50, 122.53 (122.08), 120.02, 118.18, 112.53 (110.92). HRMS-ESI (*m/z*): [M+Na]^+^ calcd. for C_13_H_9_ClN_2_Na 251.0352; found 251.0356.

*5-Bromo-2-phenyl-1H-benzo[d]imidazole* (**2d**). White solid; m.p. 202–203 °C; yield: 54%. ^1^H-NMR: δ 13.09 (br s, 0.26H), 8.18–8.16 (d, 2H, *J* = 8.0 Hz), 7.87–7.50 (m, 5H), 7.37–7.33 (m, 1H). ^13^C-NMR: δ 145.20, 142.82, 130.27, 129.54, 129.01, 126.57, 125.21, 124.70, 114.71, 113.87, 113.07. HRMS-ESI (*m/z*): [M+Na]^+^ calcd. for C_13_H_9_BrN_2_Na 294.9847; found 294.9849.

*5-Methyl-2-phenyl-1H-benzo[d]imidazole* (**2e**). White solid; m.p. 242–243 °C; yield: 67%. ^1^H-NMR: δ 12.80 (br s, 0.22H), 8.20 (m, 2H), 7.55 (m, 5H), 7.05 (m, 1H), 2.45 (s, 3H). ^13^C-NMR: δ 150.80, 141.94, 135.08, 131.53, 130.24, 129.59, 128.84, 128.01, 126.26, 123.51, 118.29, 110.86, 21.27. HRMS-ESI (*m/z*): [M+Na]^+^ calcd. for C_14_H_12_N_2_Na 231.0898; found 231.0896.

*5-Methoxy-2-phenyl-1H-benzo[d]imidazole* (**2f**). White solid; m.p. 148–150 °C; yield: 67%. ^1^H-NMR: δ 13.07 (br s, 0.13H), 8.29–8.13 (m, 2H), 7.58-7.48 (m, 4H), 7.25–7.08 (m, 1H), 7.02–7.01 (m, 1H), 3.83–3.82 (s, 3H). ^13^C-NMR: δ 156.8, 151.4, 137.4, 136.2, 130.4 (130.1), 129.4 (129.2), 127.5, 126.8, 114.2, 112.4, 94.99 (94.94), 56.4. HRMS-ESI (*m/z*): [M+Na]^+^ calcd. for C_14_H_12_N_2_NaO 247.0847; found 247.0849.

*2-Phenyl-3H-imidazo[4,5-b]pyridine* (**2g**). White solid; m.p. 283–284 °C; yield: 44%. ^1^H-NMR δ 13.48 (br s, 1H), 8.34 (dd, *J* = 4.8, 1.5 Hz, 1H), 8.25−8.21 (m, 2H), 8.02 (d, *J* = 7.5 Hz, 1H), 7.61−7.51 (m, 3H), 7.25 (dd, *J* = 8.1, 4.8 Hz, 1H). ^13^C-NMR: δ 152.32, 143.75, 135.57, 130.52, 129.57, 129.00, 126.70, 126.27, 119.16, 118.09. HRMS-ESI (*m/z*): [M+Na]^+^ calcd. for C_1__2_H_9_N_3_Na 218.0694; found 218.0697.

*2-p-Tolyl-1H-benzo[d]imidazole* (**2i**). White solid; m.p. 276–278 °C; yield: 60%. ^1^H-NMR: δ 12.83 (br s, 0.15H), 8.09–8.07 (d, 2H, *J* = 7.6 Hz), 7.65–7.53 (m, 2H), 7.37–7.35 (d, 2H, *J* = 8.0 Hz), 7.20 (m, 2H), 2.39 (s, 3H). ^13^C-NMR: δ 151.17, 143.71, 139.49, 134.74, 129.42, 127.31, 126.30, 122.23, 121.50, 118.62, 111.04, 20.87. HRMS-ESI (*m/z*): [M+Na]^+^ calcd. for C_14_H_12_N_2_Na 231.0898; found 231.0895.

*2-(4-Methoxyphenyl)-1H-benzo[d]imidazole* (**2j**). White solid; m.p. 221–223 °C; yield: 63%. ^1^H-NMR: δ 12.76 (br s, 0.11H), 8.14–8.12 (d, 2H, *J* = 8.8 Hz), 7.57 (m, 2H), 7.20–7.17 (m, 2H), 7.13–1.11 (d, 2H, *J* = 8.8 Hz), 3.85 (s, 3H). ^13^C-NMR: δ 160.59, 151.25, 143.73, 134.97, 127.98, 122.61, 122.07, 121.74, 118.43, 114.34, 111.05, 55.29. HRMS-ESI (*m/z*): [M+Na]^+^ calcd. for C_14_H_12_N_2_NaO 247.0847; found 247.0851.

*2-o-Tolyl-1H-benzo[d]imidazole* (**2k**). White solid; m.p. 206–208 °C; yield: 58%. ^1^H-NMR δ 12.64 (br s, 0.11H), 7.76–7.74 (d, 1H, *J* = 6.8 Hz), 7.62 (m, 2H), 7.39–7.37 (m, 3H), 7.23–7.21 (m, 2H), 2.62 (s, 3H). ^13^C-NMR δ 151.73, 136.91, 131.15, 129.94, 129.33, 129.22, 125.85, 121.78, 20.90. HRMS-ESI (*m/z*): [M+Na]^+^ calcd. for C_14_H_12_N_2_Na 231.0898; found 231.0901.

*2-(2-Methoxyphenyl)-1H-benzo[d]imidazole* (**2l**). White solid; m.p. 181–182 °C; yield: 64%. ^1^H-NMR δ 12.13 (br s, 0.22H), 8.35–8.32 (dd, 1H, *J* = 7.6, 1.6 Hz), 7.66–7.62 (m, 2H), 7.52–7.47 (m, 1H), 7.27–7.25 (d, 1H, *J* = 8.0 Hz), 7.21–7.19 (m, 2H), 7.15–7.11 (m, 1H), 4.04 (s, 3H). ^13^C-NMR δ 156.74, 152.87, 141.72, 141.68, 131.25, 129.70, 122.02, 129.33, 121.50, 120.85, 118.45, 117.93, 112.07, 55.74. HRMS-ESI (*m/z*): [M+Na]^+^ calcd. for C_14_H_12_N_2_NaO 247.0847; found 247.0849.

*4-(1H-Benzo[d]imidazol-2-yl)-N,N-dimethylaniline* (**2m**). White solid; m.p. 272–274 °C; yield: 70%. ^1^H-NMR: δ 12.57 (br s, 0.29H), 8.01 (d, 2H, *J* = 8.0 Hz), 7.57–7.46 (m, 2H), 7.15–7.13 (dd, 2H, *J* = 6.0, 2.8 Hz), 6.85–6.83 (d, 2H, *J* = 8.0 Hz), 3.00 (s, 6H). ^13^C-NMR: δ 152.12, 151.22, 144.01, 134.78, 127.52, 121.48, 121.16, 117.99, 117.31, 111.81, 110.60, 41.07. HRMS-ESI (*m/z*): [M+Na]^+^ calcd. for C_15_H_15_N_3_Na 260.1164; found 260.1168.

*2-(m-Tolyl)-1H-benzo[d]imidazole* (**2n**). White solid; m.p. 213–215 °C; yield: 50%. ^1^H-NMR: δ 12.88 (br s, 0.21H), 8.03 (s, 1H), 7.98–7.96 (d, 1H, *J* = 8.0 Hz), 7.65–7.54 (m, 2H), 7.47–7.43 (t, 1H, *J* = 8.0 Hz), 7.33–7.31 (d, 1H, *J* = 8.0 Hz), 7.22–7.21 (m, 2H), 2.43 (s, 3H). ^13^C-NMR: δ 151.13, 143.26, 138.15, 130.47, 129.92, 129.86, 128.83, 126.96, 123.55, 122.46, 121.64, 118.78, 111.22, 21.02. HRMS-ESI (*m/z*): [M+Na]^+^ calcd. for C_14_H_12_N_2_Na 231.0898; found 231.0899.

*2-(Naphthalen-2-yl)-1H-benzo[d]imidazole* (**2q**). White solid; m.p. 206–207 °C; yield: 48%. ^1^H-NMR: δ 13.11 (br s, 0.29H), 8.76 (s, 1H), 8.34 (d, 1H, *J =* 8.0 Hz), 8.11–8.05 (m, 2H), 8.01–7.99 (m, 1H), 7.73–7.71 (m, 1H), 7.64–7.59 (m, 3H), 7.25 (m, 2H). ^13^C-NMR: δ 151.23, 143.87, 134.96, 133.45, 132.79, 128.54, 128.42, 127.77, 127.53, 127.10, 126.91, 125.79, 123.91, 122.67, 121.76, 118.88, 111.31. HRMS-ESI (*m/z*): [M+Na]^+^ calcd. for C_17_H_12_N_2_Na 267.0898; found 267.0899. 

*2-(Thiophen-2-yl)-1H-benzo[d]imidazole* (**2r**). White solid; m.p. 341–343 °C; yield: 60%. ^1^H-NMR: δ 12.94 (br s, 0.24H), 7.83 (dd, *J* = 3.6, 0.8 Hz, 1H), 7.72 (dd, *J* = 4.8, 0.8 Hz, 1H), 7.62−7.60 (m, 1H), 7.50 (dd, *J* = 6.9, 2.1 Hz, 1H), 7.25−7.16 (m, 3H). ^13^C-NMR: δ 146.86, 143.54, 134.49, 133.59, 128.73, 128.25, 126.67, 122.61, 121.74, 118.51, 111.02. HRMS-ESI (*m/z*): [M+Na]^+^ calcd. for C_11_H_8_N_2_NaS 223.0306; found 223.0304.

*2-(Furan-2-yl)-1H-benzo[d]imidazole* (**2s**). White solid; m.p. 285–286 °C; yield: 48%. ^1^H-NMR: δ 12.92 (br s, 0.26H), 7.95 (dd, *J* = 1.8, 0.9 Hz, 1H), 7.55 (br s, 2H), 7.24−7.20 (m, 3H), 6.73 (dd, *J* = 3.3, 1.8 Hz, 1H). ^13^C-NMR: δ 145.5, 144.6, 143.5, 134.3, 122.3, 121.6, 118.7, 112.3, 111.4, 110.5. HRMS-ESI (*m/z*): [M+Na]^+^ calcd. for C_11_H_8_N_2_NaO 207.0534; found 207.0536.

*2-Methyl-1-phenyl-1H-benzo[d]imidazole* (**2u**). White solid; m.p. 127–129 °C; yield: 33%. ^1^H-NMR: δ 7.67–7.63 (m, 3H), 7.59–7.53 (m, 3H), 7.24–7.12 (m, 3H), 2.43 (s, 3H). ^13^C-NMR δ 143.2, 136.1, 134.3, 130.4, 129.2, 127.3, 124.5, 122.8, 122.4, 118.9, 110.3, 14.6. HRMS-ESI (*m/z*): [M+Na]^+^ calcd. for C_14_H_12_N_2_Na 231.0898; found 231.0896.

## 4. Conclusions

In summary, a straightforward weak base-mediated protocol had been developed for the intramolecular C–N bond formation to provide benzimidazole derivatives in moderate to high yields. Particularly interesting, the use of water as a benign and accessible solvent should render the methodology described herein economical and environmentally attractive, providing an alternative synthetic protocol for potential industrial applications without the addition of any exogenous transition metal catalysts.
